# Reflections on the Predictability of Evolution: Toward a Conceptual Framework

**DOI:** 10.1016/j.isci.2020.101736

**Published:** 2020-10-27

**Authors:** Alix Mas, Yvan Lagadeuc, Philippe Vandenkoornhuyse

**Affiliations:** 1 Université de Rennes 1, CNRS, UMR6553 ECOBIO, Campus Beaulieu, Avenue Leclerc, Rennes Cedex 35042, France

**Keywords:** Evolutionary Biology, Evolutionary Theories

## Abstract

Evolution is generally considered to be unpredictable because genetic variations are known to occur randomly. However, remarkable patterns of repeated convergent evolution are observed, for instance, loss of pigments by organisms living in caves. Analogous phenotypes appear in similar environments, sometimes in response to similar constraints. Alongside randomness, a certain evolutionary determinism also exists, for instance, the selection of particular phenotypes subjected to particular environmental constraints in the “evolutionary funnel.” We pursue the idea that eco-evolutionary specialization is in some way determinist. The conceptual framework of phenotypic changes entailing specialization presented in this essay explains how evolution can be predicted. We also discuss how the predictability of evolution could be tested using the case of metabolic specialization through gene losses. We also put forward that microorganisms could be key models to test and possibly make headway evolutionary predictions and knowledge about evolution.

## Graphical Abstract

**Figure:**
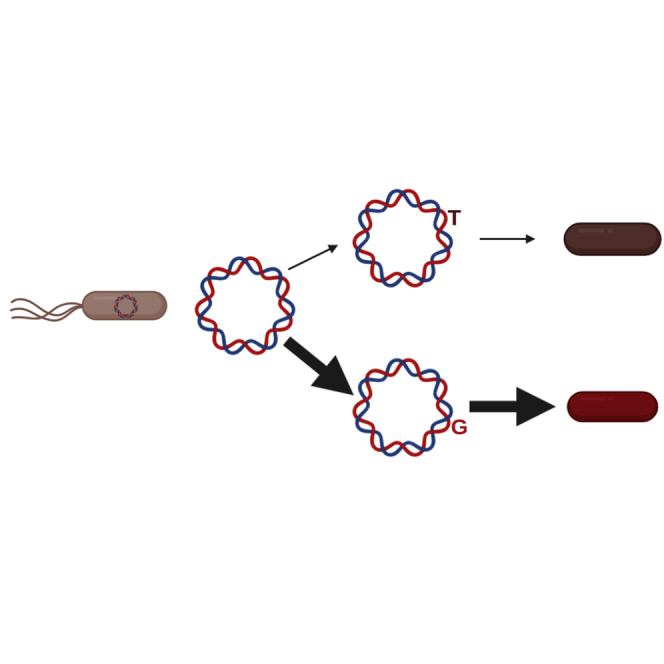

## Facing the Difficulty of Predicting Evolution

Two hundred years into the exploration of evolution and the question of predictability is still the subject of lively debate.

Beliefs constantly shift from a deterministic Lamarckist view to all-random insights of the neutral theory (Kimura, 1983), and questions of determinism, and hence of predictability, have made ink and ideas flow, as they have triggered many research studies (Blank et al., 2014; de Visser and Krug, 2014; Duarte et al., 2015; Fragata et al., 2019; Kryazhimskiy et al., 2014; Lapidot and Conley, 2015; Stern and Orgogozo, 2008; Szendro et al., 2013; Wang et al., 2018).

Today, awareness of the complexity of biological systems supports the perception that evolution is unpredictable. Indeed, any biological system is composed of so many intertwined and co-operating components that it can be considered as a “chaotic” system, of which only an omniscient and omnipotent mind could precisely predict evolution. However, it should be noted that the central tenants of Neo-Darwinism and related theoretical concepts is that organisms that are best adapted to the environment will be selected, and in a way, are thus deterministic. From this theoretical way of thinking, a study on the long-term evolution of complex phenotypic systems confirmed that stochastic-like dynamics were more likely to determine evolution (Doebeli and Ispolatov, 2014). That study, along with others (Huneman, 2012), points out that evolution may be deterministic, but is nonetheless not predictable.

**Figure 1:**
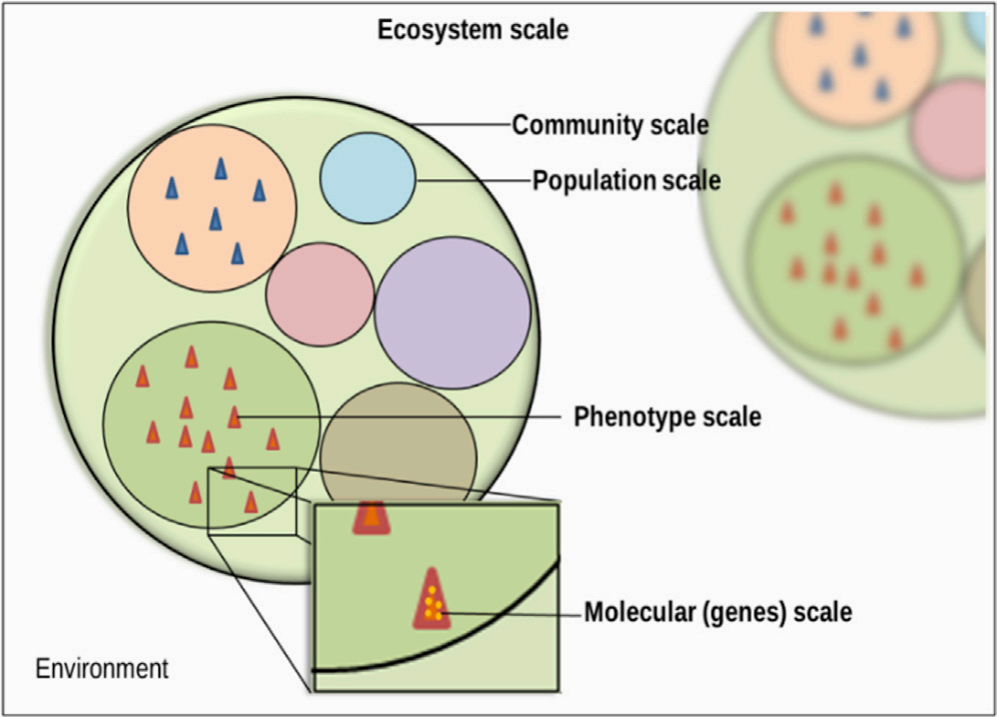
The Ecological Levels of Integration The different levels of ecological hierarchy are represented: genomic (i.e., inside the triangles), individual phenotype (i.e., a triangle) on which selection acts as engine of population dynamics (i.e., a circle), community (i.e., circle embedding populations), and ecosystems (i.e., embedding communities at a given [geographic] location). The functional level is also considered in ecology; it corresponds to the activity and roles in the ecosystem expressed by an organism or a population.

Predicting evolution is intricate as it invokes every biological level; from the modification of the molecular structure of DNA, to a change in functions and phenotypes, and even population and community structures (Figure 1). Each of these levels has its own complexity, effects, and feedbacks at other levels as they are all inextricably interwoven with each other.

### Acknowledging Randomness

Genetic variants occur randomly, as a result of errors in DNA replication that are eventually incorporated in the genome. It is thus impossible to predict where on the genome, on which nucleotides, codons, or set of genes and which type of genetic change (insertion, deletion, point mutation either synonymous or missense) will occur. If the genetic variation has a circumneutral or positive effect, then it is expected to be transmitted to the following generation if the population is large enough to limit genetic drift (random fixation of allele(s) in a population of limited size).

If they rely on the concept of fitness (optimization/maximization), accurate predictions of genetic modifications are theoretically possible. To over-simplify, if a known mutation provides better fitness to an organism (i.e., non neutral), then one can predict that this mutant will be selected for within the fitness landscape, i.e., will lead to a local or global fitness maximum of the fitness landscape (Fragata et al., 2019; Gavrilets, 2004). However, predictions concerning the emergence of new mutations per se are still extremely complex because the modification of a phenotype is not necessarily determined by a linear modification of a gene. Some genetic modifications will not affect phenotypic traits at all (neutral evolution), whereas others can have a cascade of phenotypic consequences if they occur on a regulatory sequence of a microbial operon, for instance, on pleiotropic genes (pleiotropy controls the expression of several phenotypic traits by a given gene) or on epistatic genes (simplistically defined, an epistatic gene is a gene that determines whether or not a trait will be expressed).

Different mutations may also result in a similar phenotypic trait (Bridgham, 2016; Giddins et al., 2017). Otherwise, successive modifications of a genome over time, whether neutral or not, could give rise to the emergence of a new phenotypic feature (Loeb et al., 2003). This adds to the complexity of predicting the occurrence and effects of mutations on phenotypes.

### Considering Phenotype

If we knew what traits or features would need to be adjusted to increase the fitness of an organism in a given environment (for example, changing the color of the organism to make it more cryptic and hence less prone to predation, Arendt and Reznick, 2008; Orteu and Jiggins, 2020) we would be able to predict such variations in phenotype (i.e., not flat fitness landscape from non-neutral genetic modifications).

**Figure 2:**
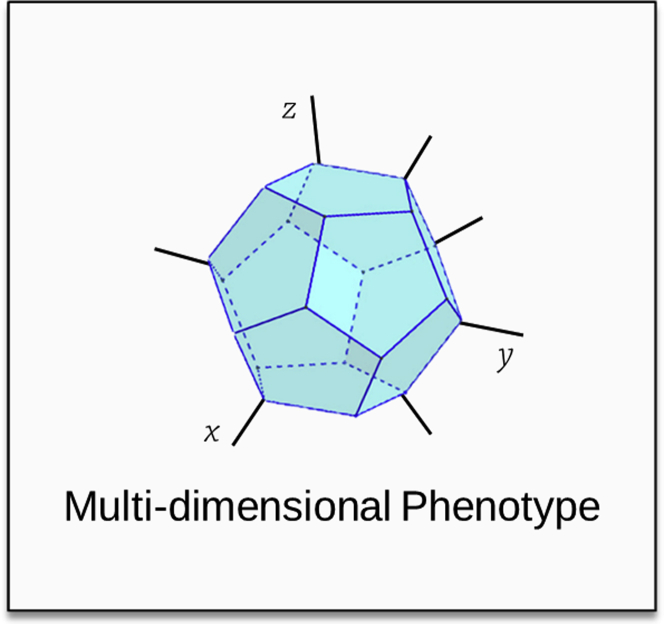
Phenotype Seen As a Hyper-volume in Multidimensional Space Each ridge of the volume is a trait of the phenotype. Ridges can take different values. Some of the traits may be related to others (functional trade-offs), and their variation will influence the variations in related traits. To facilitate interpretation, the volume is very homogeneous, but each side of the volume could be shaped and sized differently.

A phenotype is actually the combined effect of traits and functions expressed by an organism. Phenotypes can be theorized as multi-dimensional systems (Figure 2) in which a phenotypic modification is often the result of the evolution of multiple traits. This theorized multidimensional system can be seen as the result at a given time point of the fitness optimization toward a local or a global maximum within the fitness landscape. We suggest that assessing the evolution of one particular trait might be far from the actual constraints that affect the whole organism. For example, we cannot necessarily determine which trade-offs such modifications would involve, which functions would be diminished or impaired, and what evolutionary consequences it could have.

Understanding the evolutionary dynamics of a single trait is interesting but likely an incomplete perception and oversimplification of the evolution of the organism, making accurate evolutionary predictions of this trait difficult.

### Environmental Parameters

In addition to these straightforward limits to predicting evolution, it is also important to underline that, like phenotypes, the environment is a dynamic and multidimensional system. Consequently, focusing on one or a few environmental parameters at a given time point is somewhat restricted and biases one's view of the systems.

Interactions between variables are often overlooked, and it is assumed that it is impossible to incorporate all existing variables and their interactions in *in vitro* experiments or *in silico* models (Morozov, 2013). At an evolutionary timescale, which differs depending on the organism's generation time, it is not possible to predict environmental variations with certitude, and hence the traits that organisms will develop to adequately respond to these changes.

Furthermore, the local environments are also modified by the organisms themselves as they evolve. This eco-evolutionary feedback (Ferriere and Legendre, 2013; Fussmann et al., 2007) implies the need for continual refinement of the new adaptations.

Both bottom-up (genome to phenotype) and top-down (environment to phenotype) considerations suggest that, due to their respective randomness and complexity, forecasting the precise emergence of a multi-dimension phenotype (Figure 2) in a given environment is out of reach. In addition, there is an important corpus of knowledge indicating that adaptation rates are tightly dependent on both mutation rates (or other vertically transmitted genetic innovation) and population sizes (e.g., Desai et al., 2007; Lang et al., 2011).

## Facing the Obviousness of Convergent and Repeated Evolution

### Convergence of Phenotypic Features

When narrowed down to simpler systems, distinct evolutionary patterns emerge, suggesting the existence of constrained mechanisms.

Remarkable patterns of repeated evolution have been observed at the phenotypic level, i.e., where analogous phenotypes appear in similar environments, sometimes to respond to similar constraints (Arendt and Reznick, 2008; Bailey et al., 2017; Conway Morris, 2010; Gompel and Prud'homme, 2009; Lee and Marx, 2012).

At the phenotypic level, organisms living in similar environments continually evolve the same features. This phenomenon has been widely studied through the evolutionary radiations of cichlid fish in the African Rift lakes (Muschick et al., 2012; Sturmbauer et al., 2010), in stickleback fish (Rundle, 2000), or through the radiation of *Anolis* lizards (Losos, 1998), where in each case, a set of similar phenotypes emerged in the ecosystem to which they were subjected. This kind of repeated evolution is also apparent, for example, in the emergence of similar environmentally coherent coloration-phenotypes in mice and fish (Comeault et al., 2016; Gompel and Prud'homme, 2009). Parallel evolution also operates at the functional level, as demonstrated by the repeatable experiments of Rainey and Travisano (1998), in which a population of bacteria repeatedly evolves several phenotypes to better exploit the experimental environment. Herron and Doebeli (2013) thus argued that [ …] *parallel genetic changes underlying similar phenotypes in independently evolved lineages provide empirical evidence of adaptive diversification as a predictable evolutionary process* [ …]. The fact that similar phenotypes emerged in distinct space and time, and repeatedly in either closely related or highly different species (Bridgham, 2016; Gompel and Prud'homme, 2009) suggests that certain types of transformation are favored in evolution. However, it has also to be underlined that the fate of particular beneficial mutations in independent populations of yeast strains was very dependent on other mutations and “background” genetic variation (Lang et al., 2011), i.e., an epistasis phenomenon where an initial mutation contingents future evolution (e.g., Blount et al., 2008; Kryazhimskiy et al., 2014; Jerison and Desai, 2015).

Beyond the neutral theory, it may be possible that some constraints, for example. at the molecular or metabolic levels, effectively shape potential outcomes, leading to the emergence of a finite set of phenotypes that fit the environment, and among these possibilities, the one selected is the one that provides the optimal response, often (but not always) resulting in the emergence of similar features in organisms.

From all these accumulated observations and even without necessarily knowing the cause of phenotypic convergence, predicting evolution at the phenotypic level sounds reasonable for certain environments.

### Observed Emergence of Similar Genetic Changes

On a relatively frequent basis, the emergence of analogous phenotypic features is driven by similar genetic modifications that arose independently. This is the case for the mutation of genes that encode pigmentation shared by very different species and that mutate to cause loss of pigmentation in caves (Gompel and Prud'homme, 2009; Gross et al., 2009) in birds; the convergent evolution of hemoglobin to adapt to altitude (Natarajan et al., 2016); in a variety of insects, the development of resistance to the toxicity of plants they consume (Dobler et al., 2012); in snakes, resistance to poison (Feldman et al., 2012); in *Bacillus* spp., evolution of a compensatory mutation to reduce the cost of antibiotic resistance (Levin et al., 2000); in plants such as *Amaranthus tuberculatus*, the evolution of resistance to glyphosate herbicides (Kreiner et al., 2019); and in fish species, the spectacular recurrent missense mutation in rhodopsin to adapt to light conditions in the Baltic Sea (Hill et al., 2019), to mention only a few of the compelling examples across different domains of life.

This parallel evolution at the genetic level emerged either because (1) the same families of genes (Giddins et al., 2017), orthologous genes (genes of identical origin), were modified (Kreiner et al., 2019), and even (2) the same nucleotides on a given gene were affected (Bailey et al., 2017; Hill et al., 2019; Rosenblum et al., 2014; Signor et al., 2016).

At the genetic level, clear types of repeated events are reported. Regions of the genomes more susceptible to mutate than others are well described (Stern, 2013). Some genes are known to mutate more often than others, as is the case for duplicated genes (Toll-Riera et al., 2016). For other genes, it could be their position in the gene regulatory network or their pleiotropic effects that determine their propensity to mutate (Gompel and Prud'homme, 2009).

The frequency of convergent evolution is sufficiently conspicuous to make one wonder if evolution is truly a fully stochastic process. Yet one must also keep in mind that it is virtually impossible to detect mutations that are not conserved because they do not improve fitness or are maladaptive. Thus, from a static viewpoint, it appears that some genes are repeatedly modified, whereas it could be that other genes were also mutated but the changes were not conserved by natural selection. In that sense, it is difficult to know if mutations are random or constrained molecular events, and there are perhaps still some unraveled mechanisms for which, currently, we only see the outcome as random. Indeed the convergence and repeatability of observed features (Hill et al., 2019; Natarajan et al., 2016; Rosenblum et al., 2014; Stern, 2013) suggest that genetic modifications are constrained (to optimize the effect of mutations with the fewest possible collateral effects) and thus restricted to a subset of solutions. Yet these conspicuous patterns could be explained by the fact that the solutions we can see are the ones that result in the overall fittest phenotypes, and are thus selected for.

Even if today it still seems impossible to accurately forecast the occurrence of a precise mutation on a single nucleotide to determine adaptative evolution, as described above, recent advances in our understanding of the recurrence of mutations and gene modification patterns suggest mechanisms as yet unraveled that would enhance predictability. We propose hereafter a new framework to better apprehend and predict evolutionary trajectories especially by working with microorganisms to take advantage of their time of generation and thus to experiment evolution. Of course, behind this framework, any kind of genetic changes transmittable to the next generation have to be considered, which thus also encompasses horizontal gene transfers (Ochmam et al., 2000) within clonal bacterial populations. Conversely, genetic events inducing particular fitness patterns in population dynamics, as genetic hitchhiking, are not considered. Population size and especially bottlenecks, which often exist in published articles, are not considered either.

## Framework Favoring Evolutionary Predictions

Despite the stochasticity attributed to evolution processes (i.e., random mutation and genetic changes, genetic drift acting on populations), the repeated emergence of similar gene modifications and of identical phenotypes under similar environmental constraints suggests that evolution is at least partly shaped by constraints at different biological levels (constraints on the functioning of the genome, functional trade-offs, biotic and abiotic environments) that narrow the set of alternative evolutionary trajectories that organisms can follow that reduce the number of achievable optimal solutions.

### The “Evolutionary Funnel” as a Driver of Convergent Evolution

**Figure 3:**
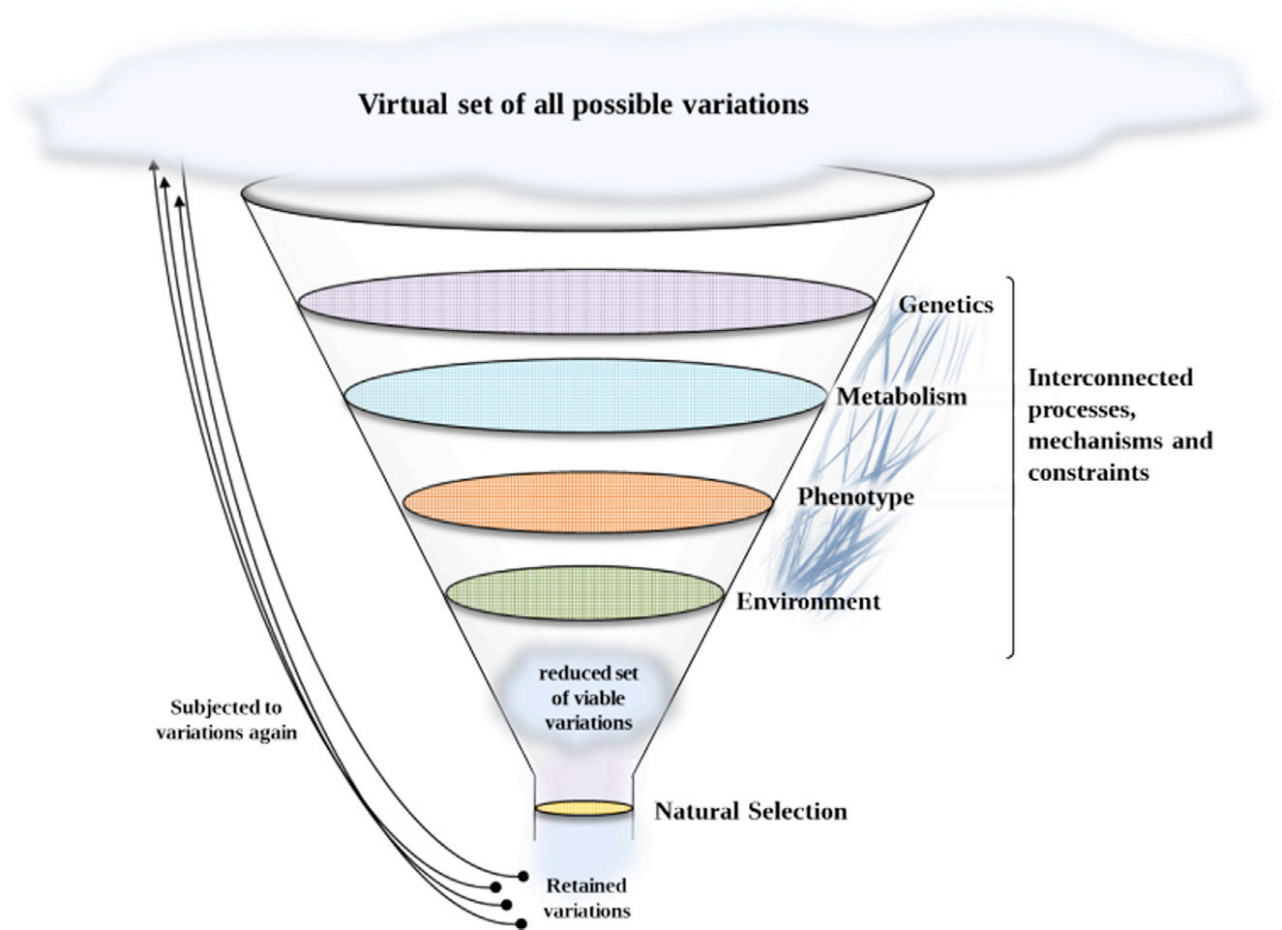
The “Evolutionary Funnel” Showing the Constraints That Shape Evolutionary Possibilities The first level of constraint is the intrinsic (physicochemical) properties of the genetic code that enable only restricted modification of the genome. Genome-wise, the complex network of interacting genes limits possible modifications, as any modification in a gene can have a cascade of effects on other genes. Here, the effect of pleiotropic genes is crucial as the constraints exerted on these genes are strong. The changes also have to be viable, with their core metabolic functions conserved (not carrying important modifications), whereas other accessory functions will be more readily modified (Lee and Marx, 2012). Trade-offs at phenotypic levels will also shape the possible evolutionary trajectory. Finally, the biotic and abiotic environment also constrains evolution and thus population dynamics, for example, through the available resources and the interacting species present, thereby modifying population and community dynamics. Natural selection (i.e., adaptation to the existing environment) will drive the conservation of particular adaptive solution(s).

Very often, only the environment is considered as a filter that enables organisms to thrive or leads to their decline (i.e., phenotypes are “sieved” and conserved or ultimately discarded). However, every biological level (genetic, metabolic, functional, etc.) of a living system is constrained in some way, which reduces the set of valid possibilities for adaptive evolution (Figure 3). The conceptual “evolutionary funnel” (Figure 3) explains how starting from the virtual set of potential modifications that could emerge at the individual level, these variations are “sieved” by the different constraints that exist at the different biological levels.

Environmental constraints act on the expressed phenotypes, the phenotype being one consequence of genome expression and functioning, itself determined by genetic variations (Pelletier, 2019). From this conceptual understanding, the predictability of evolution (and hence related retained genetic changes) is rooted in understanding the consequences of the environmental filtering of the expressed phenotypes. In this “evolutionary funnel,” the possibility of convergent traits can be triggered both by similar genetic modifications and underlying molecular events or by changes in dissimilar genes. The accuracy of the predictability is likely to be positively linked with the stringency of the constraints.

Evolution is therefore the result of several different forces and constraints at each biological level considered (Figure 1). The virtual set of every potential recombination, mutation, and phenotype that could come into existence is reduced to a subset of possibilities, sometimes leading to the emergence of similar evolutionary trajectories.

### Evolution of Specialization

As defined by the “evolutionary funnel” (Figure 3), organisms are subjected to multi-factorial (and dynamic) constraints that are both intrinsic and environmental. The organisms search for adaptive compromises (trade-offs) to balance each of these constraints. Of course, these constraints are acting on all the organisms thus on populations dynamics.

**Figure 4:**
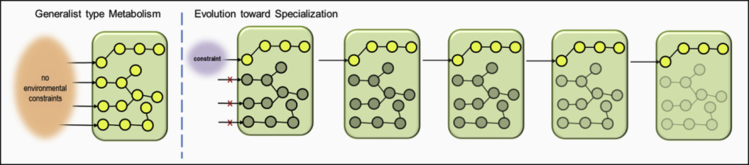
Simplified Representation of the Metabolic Optimization Concept The “metabolic streamlining” hypothesis (Giovannoni, 2005; Tripp et al., 2010) is represented in a simplified way. The green rectangle represents the organism, the circles and lines schematically represent the metabolic network of the organism, and the arrows represent nutrient uptake. The yellow circles represent “activated” metabolic pathways, and the gray circles, inactivated pathways. In an environment with no constraints (left), organisms can exploit all the nutrients present (if they have the necessary capacity) and the corresponding metabolic pathways will consequently be activated. Conversely, in an environment in which the nutrient resources are constrained, say, to one-carbon source, only the metabolic pathway that is activated will be essential. The other unused pathways could, after some evolutionary time and according to streamlining hypotheses, decay (i.e., the related genes will no longer be under selection), leading to the specialization of the metabolism of the organisms. This channeled evolution trajectory is probably predictable and experimentally testable.

We are assuming there is a higher potential for prediction of evolutionary trajectories in a theoretically “reduced” environment (with either fewer variables than the original environment, or variables with a smaller range of variation) than in an “expanded” environment with new types of variables. To give an example, let us consider only one metabolic function of an organism (e.g., nutrient uptake), and that the organism has the capacity to “display'” this function in 10 variations, for instance, the uptake of 10 different types of nutrients. Let us imagine that only one nutrient, always the same, is available. We can predict that this environmental pressure on population and community dynamics would lead to specialization toward this particular nutrient, and thus to metabolic optimization (Figure 4). Reciprocally, it seems difficult to predict an adaptative innovation enabling the uptake of an 11^th^ nutrient (Figure 4).

Such reductive specialization of organisms to their (biotic and abiotic) environments is not rare. It has been studied in depth in both endosymbionts (Lai et al., 1994) and free-living microorganisms (Boscaro et al., 2013; Giovannoni, 2005; Swan et al., 2013). As long as the environmental constraints are strong and sufficiently stable over evolutionary time, it can give rise to specialization (the term specialization hereafter includes “reductive” evolution of functions such as the loss of function(s) compared with its former capacities).

In stable environments, organisms are known to specialize, if specialization confers fitness benefits, which thus impact the population dynamics. Specialization involving adaptation to particular environmental constraints is regularly associated with genome reduction in microorganisms (Dufresne et al., 2005; Morris et al., 2012). In short, such a reduction can happen either through selection for reduction (i.e., superfluous and costly functions “*have to be”* deleted) or more likely because functions that are superfluous in a given environment undergo a lift of selection and the genes involved in these functions are consequently fated to decay through the accumulation of mutations (Lahti et al., 2009). The notions of “selection for” or “lift of selection” at the genetic level can be explained by the fact that adaptive trade-offs occur at the metabolic level.

**Figure 5:**
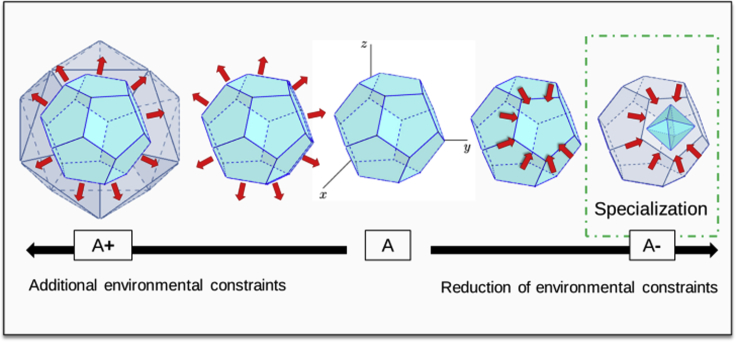
Conceptual Framework of Phenotypic Changes Leading to Specialization (A) The central hypervolume is a simplified representation of the phenotype of an organism; each ridge of the volume is a feature of the organism that can display variable values (for example, a ridge is the “color of the organism,” which can take the values beige, gray, brown, black, etc.). (A+) The expanding space of possibilities on the left (gray space of the volume) suggests that if an organism changes to a new environment with previously inexperienced parameters, it may have to develop new features, or new variations of existing features that are not part of the current phenotype (which could happen through gene duplication or gene acquisition via horizontal transfer). In this context, prediction is difficult, as one would have to identify all possible innovations and evolutionary trajectories. (A-) The reduced volume schematizes the specialization of an organism when its environment is reduced, more constrained in the range of existing parameters. In this case, predicting evolutionary trajectories and population dynamics toward a maximized local or global fitness is within the realm of possibility as we expect already existing features to be modified to optimize their activity. For example, features that enable a response to a constraint that is conserved should be enhanced, whereas features that enabled a response to constraints that were removed could decay.

Generalists can be predicted to be favored and to emerge in a switching environment (Wang and Dai, 2019). A change in metabolism is one of the first response of organisms in adapting to new or changing environments (Wang and Dai, 2019; Lopatkin and Collins, 2020). Nevertheless, in a switching environment, organisms may have to develop new features and traits in a highly complex space of evolutionary possibilities (Wang and Dai, 2019), which thus seems very difficult to predict (A+ in Figure 5).

Reciprocally, in a temporally stable and spatially homogeneous environment, the activity of some metabolic functions will be favored over others, leading to the differential expression of the genes involved in these functions. In this stable environment, metabolic optimization (Figure 4) is expected to be progressively integrated in the constitutive genome during specialization as the organisms will benefit from specializing in response to the environment.

It is expressly in the context of evolution toward specialization (conforming with a “reduced” environment compared with the original environment) that it is probably feasible to prune the space of evolutionary possibilities and thus to predict, at least to some extent, the evolutionary trajectories that will be followed by the organism at the genetic and population levels (A- in Figure 5). If the phenotypic changes that entail specialization (Figure 5) are predictable, for instance, the convergent loss of pigmentation and vision by animals living in caves, the related genetic changes might also be predictable even if not necessarily the only solution that could lead to the expected phenotypic change.

## How to Approach Predictions with Microorganisms?

Advances in sequencing technologies have made it possible to analyze the genomes of populations/communities that have experimentally evolved and to test evolutionary-based hypotheses that have blossomed in the recent years, primarily by using microorganisms as model (e.g., Jerison and Desai, 2015). In this way, the famous long-term evolution experiment with *Escherichia coli* (Lenski et al., 1991; Barrick et al., 2009; Barrick and Lenski, 2013) enabled *replaying life's tape* (Blount et al., 2018) and showed that the trait's evolution was dependent on the prior occurrence of particular mutations. This epistasis phenomenon has also been nicely demonstrated in experimental yeast evolution of populations (Kryazhimskiy et al., 2014) with convergent fitness evolution pattern in laboratory conditions even if sequence-level adaptation appeared stochastic. Parallel experiments of evolution in 120 separated *E. coli* populations grown at high temperatures has also made it possible to assess the frequency of each single mutation within and among populations and to estimate their frequency relative to a null random model of mutation accumulation to highlight among others this epistasis (Tenaillon et al., 2016). It would also be possible to assess bias toward non-synonymous mutations, parallelism among the accumulated mutations in parallel evolution experiments (Bruger and Marx, 2018), evolutionary trajectories, and their associated functional consequences. Unless the existence of epistasis, i.e., the contingency applied on the fate of a genetic innovation by previous evolutionary steps, predictive biology on microorganisms is developing (Lopatkin and Collins, 2020). This predictive biology, rooted in systems biology, makes the interpretation of genes within models of intricate circuits (e.g. metabolic pathways for instance). This way of seeing living microorganisms allows to model the genome functioning. Under this framework, microbial population dynamics in controlled or natural systems relies on intrinsic processes related to individual genome and genome expression, and also on eco-evolutionary extrinsic factors (Lopatkin and Collins, 2020), hypothesized herein under the view of the evolutionary funnel (Figure 3). Under this view, the reductive specialization of organisms submitted to a continuous constraint (Figure 4) could permit to predict the positive selection for the used metabolic circuits (i.e., acting on individuals of a given population) and conversely the negative selection on the unused set of genes leading to pseudogenes (i.e., inactivated genes by genetic alteration), and ultimately, gene-loss(es) by neutral ratchet-like loss (e.g., Wolf and Koonin, 2013).

### Evolution and Co-evolution of Metabolism in Free-Living Microbial Populations and Communities

#### Interactions between Free-Living Microorganisms

Metabolism can influence interactions between organisms and can even shape co-evolution between related or distant species. The same goes for endosymbiotic relationships, like the relationship between aphids and *Buchnera aphidicola* (i.e., for a review on genome streamlining, Lynch 2006), and also for free-living organisms, as in the evolution of dependencies based on the production of a common good allowing the loss of common good production gene(s) (e.g., Black Queen Hypothesis, Morris et al., 2012; Mas et al., 2016).

Free-living microorganisms excrete compounds and enzymes that control relationship with other individuals or populations. As a trivial example, if in a toxic environment, a microbial population emits a detoxifying enzyme, it can be predicted that variants no longer able to produce this detoxifying enzyme will emerge and become dependent on the detoxifiers if this functional loss provides a fitness gain (i.e., energy saved that is reallocated to survival and reproduction) leading to a population-level selection (Mas et al., 2016). The same process is expected to occur at the community level (Mas et al., 2016) in both cases, leading to a population-level steady state. In these cases, the loss of function can be considered as an eco-evolutionary process of specialization (i.e., niche reduction) enabling the organism to escape competition and optimizing the use of available resources. In the specific context of specialization leading to a niche reduction of the evolved population (i.e., specialization toward the exploitation of particular environmental resources), it would thus be possible to infer which metabolic function and pathways are essential and which are of little use.

#### Predicting and Testing an Evolution Trajectory

Based on current knowledge, we can theoretically predict which genes will not be needed under specific living constraints and thus likely to accumulate genetic changes (i.e., decay). For example, in the context in which an organism is able to metabolize many different carbon sources, but is subjected to only one of the carbon sources, we would expect the organism to specialize in the consumption of the specific resource, and superfluous pathways involved in the use of other carbon sources to progressively decay (Figure 4). Of course, it is the fate of newly formed pseudogenes within the populations that have to be analyzed and interpreted (e.g., Lang et al., 2013).

A metabolic approach to modeling is thus expected to improve the characterization of the constraints that shape evolutionary trajectories, at both phenotypic and ecological levels. It could also make it possible to directly link environmental constraints to potential genetic modifications by considering mutations under negative or positive selection (Lässig et al., 2017).

Concerning this evolutionary specialization trajectory toward a reduced niche, we are assuming that genes under strong selection, and those that are not under selection, can be predicted to display particular genetic modifications (e.g., stop codon mutation, indels, within unused genes thus forming pseudogenes). Modeling strategies such as flux analysis make it possible to define metabolic functions, depending on the environmental variables used as inputs in the model. In this way, the metabolism of specialized individual(s) (i.e., optimized solution(s)) can be identified from the generalist microorganism ancestor genome, and the metabolic pathways preferentially activated and, reciprocally, inactivated, can be predicted. Genome scale modeling approaches as flux balance analysis (FBA), flux variability analysis (FVA) and others that make it possible to apply conditional and stoichiometric information including the presence-absence of particular resources in the environment, that can be used to model the molecular physiology of an organism under the hypothesis of optimal genome functioning for biomass production given the growth conditions, and also machine-learning approaches for weighting and predicting metabolic costs (Wu et al., 2016). Dedicated evolution experimental design using this generalist bacterium would make it possible to test the accuracy of the predictions (e.g., stop codon mutation within unused genes, high positive selection for particular functions, and related genes) after sequencing. This strategy of combining modeling and experimentation of evolutionary trajectories might also enable improvement of the modeling approaches to provide more accurate predictions and to better understand the limits of evolution predictability.

## Conclusions

We identified different challenges linked to the prediction of evolutionary scenarios. (1) Knowing the fittest features of an organism does not necessarily enable us to know which gene should be modified and (2) the links between the selected features and genes are not necessarily straightforward (e.g., behavioral modification leading to improved features; epistasis; epigenetic mechanisms). Because all the predictions will be based on basic knowledge (e.g., good annotated genome(s), deep knowledge of the gene functions vehicled), all predictions are made under the assumption that the information is accurate and unbiased. All predictions are made under the hypothesis of individual fitness optimization/maximization. Future approaches of microbial genome and transcriptome single-cell analyses (Rosenthal et al., 2017) might provide valuable information to better assess changes to the population and to the microbial community over time and to better assess the “life-tape,” and, based on this information, to better assess the determinism and stochastic processes, improving both the modeling methods and knowledge.

Herein, we focused on microorganisms because they have been shown to be useful in evolution experiments thanks to their short generation time, and also because they are asexual. A better understanding of to what extent evolution is predictable could obviously affect our perception of evolutionary theory, and also more broadly, our perception of life and possibly our usages, the use of antibiotics, for instance. If evolution is predictable, predictable evolutionary scenarios and predictive biology will have to be taken into account in many fields including the evolutionary consequences of uses for more sustainable agriculture and a more holistic view of human health. As emphasized by Lässig et al. (2017) future research [ …] *will show how predictability plays out in more complex systems, including populations with various rates of recombination* [ …].

Exploration of evolution has been going on for more than a century, yet it is still developing, with an ever more fine-tuned understanding and conceptualization of the mechanisms underlying its functioning. Models, experiments, and sequencing technologies provide all the necessary tools to test and possibly validate evolution predictability (Lopatkin and Collins, 2020).

The cross-disciplinary lines of evolutionary studies enable an integrative and detailed view of evolutionary mechanisms. As this deep understanding is reached, repeatable and convergent configurations are emerging, reflecting potentially predictable patterns of evolution at different levels of integration. As Stephen Jay Gould proposed in his book, [ …] if we were to rewind the clock of evolution, it would probably give rise to something completely different [ …] (Gould, 1990). Using contemporary organisms, one day we might also be able to contemplate the future evolutionary options before the tape is recorded and possibly intervene to avoid a cul-de-sac and possibly also to prevent the consequences of anthropogenic pressures.

## References

[bib1] Arendt J., Reznick D. (2008). Convergence and parallelism reconsidered: what have we learned about the genetics of adaptation?. Trends Ecol. Evol..

[bib2] Bailey S.F., Blanquart F., Bataillon T., Kassen R. (2017). What drives parallel evolution?: how population size and mutational variation contribute to repeated evolution. BioEssays.

[bib3] Barrick J.E., Yu D.S., Yoon S.H., Jeong H., Oh T.K., Schneider D., Lenski R.E., Kim J.F. (2009). Genome evolution and adaptation in a long-term experiment with *Escherichia coli*. Nature.

[bib4] Barrick J.E., Lenski R.E. (2013). Genome dynamics during experimental evolution. Nat. Rev. Genet..

[bib5] Blank D., Wolf L., Ackermann M., Silander O.K. (2014). The predictability of molecular evolution during functional innovation. Proc. Natl. Acad. Sci. U S A.

[bib6] Blount Z.D., Borland C.Z., Lenski R.E. (2008). Historical contingency and the evolution of a key innovation in an experimental population of *Escherichia coli*. Proc. Natl. Acad. Sci. U S A.

[bib7] Blount Z.D., Lenski R.E., Losos J.B. (2018). Contingency and determinism in evolution: replaying life’s tape. Science.

[bib8] Boscaro V., Felletti M., Vannini C., Ackerman M.S., Chain P.S.G., Malfatti S., Vergez L.M., Shin M., Doak T.G., Lynch M., Petroni G. (2013). Polynucleobacter necessarius, a model for genome reduction in both free-living and symbiotic bacteria. Proc. Natl. Acad. Sci. U S A.

[bib9] Bridgham J.T. (2016). Predicting the basis of convergent evolution. Science.

[bib72] Bruger E.L., Marx C.J. (2018). A decade of genome sequencing has revolutionized studies of experimental evolution. Current Op. Microbiol..

[bib10] Comeault A.A., Carvalho C.F., Dennis S., Soria-Carrasco V., Nosil P. (2016). Color phenotypes are under similar genetic control in two distantly related species of *Timema* stick insect: genetic architecture of color in *timema*. Evolution.

[bib11] Conway Morris S. (2010). Evolution: like any other science it is predictable. Phil. Trans. R. Soc. B.

[bib12] de Visser J.A.G.M., Krug J. (2014). Empirical fitness landscapes and the predictability of evolution. Nat. Rev. Genet..

[bib13] Desai M.M., Fisher D.S., Murray A.W. (2007). The speed of evolution and maintenance of variation in asexual populations. Curr. Biol..

[bib14] Dobler S., Dalla S., Wagschal V., Agrawal A.A. (2012). Community-wide convergent evolution in insect adaptation to toxic cardenolides by substitutions in the Na,K-ATPase. Proc. Natl. Acad. Sci. U S A.

[bib15] Doebeli M., Ispolatov I. (2014). Chaos and unpredictability in evolution: chaos and unpredictability in evolution. Evolution.

[bib16] Duarte J., Rodrigues C., Januário C., Martins N., Sardanyés J. (2015). How complex, probable, and predictable is genetically driven red queen chaos?. Acta Biotheor..

[bib17] Dufresne A., Garczarek L., Partensky F. (2005). Accelerated evolution associated with genome reduction in a free-living prokaryote. Genome Biol..

[bib18] Feldman C.R., Brodie E.D., Brodie E.D., Pfrender M.E. (2012). Constraint shapes convergence in tetrodotoxin-resistant sodium channels of snakes. Proc. Natl. Acad. Sci. U S A.

[bib19] Ferriere R., Legendre S. (2013). Eco-evolutionary feedbacks, adaptive dynamics and evolutionary rescue theory. Phil. Trans. R. Soc. B.

[bib20] Fragata I., Blanckaert A., Dias Louro M.A., Liberles D.A., Bank C. (2019). Evolution in the light of fitness landscape theory. Trends Res. Ecol. Evol..

[bib21] Fussmann G.F., Loreau M., Abrams P.A. (2007). Eco-evolutionary dynamics of communities and ecosystems. Funct. Ecol..

[bib22] Gavrilets S. (2004). Fitness Landscapes and the Origin of Species, Monographs in Population Biology.

[bib23] Giddins M.J., Macesic N., Annavajhala M.K., Stump S., Khan S., McConville T.H., Mehta M., Gomez-Simmonds A., Uhlemann A.-C. (2017). Successive emergence of ceftazidime-avibactam resistance through distinct genomic adaptations in *bla*_KPC-2_ -harboring *Klebsiella pneumoniae* sequence type 307 isolates. Antimicrob. Agents Chemother..

[bib24] Giovannoni S.J. (2005). Genome streamlining in a cosmopolitan oceanic bacterium. Science.

[bib25] Gompel N., Prud’homme B. (2009). The causes of repeated genetic evolution. Dev. Biol..

[bib26] Gould S.J. (1990). Wonderful Life: The Burgess Shale and the Nature of History.

[bib27] Gross J.B., Borowsky R., Tabin C.J. (2009). A novel role for Mc1r in the parallel evolution of depigmentation in independent populations of the cavefish Astyanax mexicanus. PLoS Genet..

[bib28] Herron M.D., Doebeli M. (2013). Parallel evolutionary dynamics of adaptive diversification in Escherichia coli. PLoS Biol..

[bib29] Hill J., Enbody E.D., Pettersson M.E., Sprehn C.G., Bekkevold D., Folkvord A., Laikre L., Kleinau G., Scheerer P., Andersson L. (2019). Recurrent convergent evolution at amino acid residue 261 in fish rhodopsin. Proc. Natl. Acad. Sci. U S A.

[bib30] Huneman P. (2012). Determinism, predictability and open-ended evolution: lessons from computational emergence. Synthese.

[bib31] Jerison E.R., Desai M.M. (2015). Genomic investigations of evolutionary dynamics and epistasis in microbial evolution experiments. Curr. Op. Genet. Dev..

[bib32] Kimura M. (1983). The Neutral Theory of Molecular Evolution.

[bib33] Kreiner J.M., Giacomini D.A., Bemm F., Waithaka B., Regalado J., Lanz C., Hildebrandt J., Sikkema P.H., Tranel P.J., Weigel D. (2019). Multiple modes of convergent adaptation in the spread of glyphosate-resistant *Amaranthus tuberculatus*. Proc. Natl. Acad. Sci. U S A.

[bib34] Kryazhimskiy S., Rice D.P., Jerison E.R., Desai M.M. (2014). Global epistasis makes adaptation predictable despite sequence-level stochasticity. Science.

[bib35] Lahti D.C., Johnson N.A., Ajie B.C., Otto S.P., Hendry A.P., Blumstein D.T., Coss R.G., Donohue K., Foster S.A. (2009). Relaxed selection in the wild. Trends Res. Ecol. Evol..

[bib36] Lai C.Y., Baumann L., Baumann P. (1994). Amplification of trpEG: adaptation of Buchnera aphidicola to an endosymbiotic association with aphids. Proc. Natl. Acad. Sci. U S A.

[bib37] Lang G.I., Botstein D., Desai M.M. (2011). Genetic variation and the fate of beneficial mutations in asexual populations. Genetics.

[bib38] Lang G.I., Rice D.P., Hickman M.J., Sodergren E., Weinstock G.M., Botstein D., Desai M.M. (2013). Pervasive genetic hitchhiking and clonal interference in forty evolving yeast populations. Nature.

[bib39] Lapidot I., Conley D.W. (2015). Evolution predictability, lamarck, altshuller, Darwin and chaos. Proced. Eng..

[bib40] Lässig M., Mustonen V., Walczak A.M. (2017). Predicting evolution. Nat. Ecol. Evol..

[bib41] Lee M.-C., Marx C.J. (2012). Repeated, selection-driven genome reduction of accessory genes in experimental populations. PLoS Genet..

[bib42] Lenski R.E., Rose M.R., Simpson S.C., Tadler S.C. (1991). Long-term experimental evolution in *Escherichia coli*. I. Adaptation and divergence during 2,000 generations. Am. Nat..

[bib43] Levin B.R., Perrot V., Walker N. (2000). Compensatory mutations, antibiotic resistance and the population genetics of adaptive evolution in bacteria. Genetics.

[bib44] Loeb L.A., Loeb K.R., Anderson J.P. (2003). Multiple mutations and cancer. Proc. Natl. Acad. Sci. U S A.

[bib45] Lopatkin A.J., Collins J.J. (2020). Predictive biology: modelling understanding and harnessing microbial complexity. Nat. Rev. Microbiol..

[bib46] Losos J.B. (1998). Contingency and determinism in replicated adaptive radiations of island lizards. Science.

[bib47] Lynch M. (2006). Streamlining and simplification of microbial genome architecture. Annu. Rev. Microbiol..

[bib48] Mas A., Jamshidi S., Lagadeuc Y., Eveillard D., Vandenkoornhuyse P. (2016). Beyond the black queen hypothesis. ISME J..

[bib49] Morozov A. (2013). Modelling biological evolution: recent progress, current challenges and future direction. Interf. Focus.

[bib50] Morris J.J., Lenski R.E., Zinser E.R. (2012). The black queen hypothesis: evolution of dependencies through adaptive gene loss. mBio.

[bib51] Muschick M., Indermaur A., Salzburger W. (2012). Convergent evolution within an adaptive radiation of cichlid fishes. Curr. Biol..

[bib52] Natarajan C., Hoffmann F.G., Weber R.E., Fago A., Witt C.C., Storz J.F. (2016). Predictable convergence in hemoglobin function has unpredictable molecular underpinnings. Science.

[bib53] Ochmam H., Lawrence J.G., Groisman E.A. (2000). Lateral gene transfer and the nature of bacterial innovation. Nature.

[bib54] Orteu A., Jiggins C.D. (2020). The genomics of coloration provides insights into adaptive evolution. Nat. Rev. Genet..

[bib55] Pelletier F. (2019). Testing evolutionary predictions in wild mice. Science.

[bib56] Rainey P.B., Travisano M. (1998). Adaptive radiation in a heterogeneous environment. Nature.

[bib57] Rosenblum E.B., Parent C.E., Brandt E.E. (2014). The molecular basis of phenotypic convergence. Annu. Rev. Ecol. Evol. Syst..

[bib58] Rosenthal K., Oehling V., Dusny C., Schmid A. (2017). Beyond the bulk: disclosing the life of single microbial cells. FEMS Microbiol. Rev..

[bib59] Rundle H.D. (2000). Natural selection and parallel speciation in sympatric sticklebacks. Science.

[bib60] Signor S.A., Liu Y., Rebeiz M., Kopp A. (2016). Genetic convergence in the evolution of male-specific color patterns in Drosophila. Curr. Biol..

[bib61] Stern D.L. (2013). The genetic causes of convergent evolution. Nat. Rev. Genet..

[bib62] Stern D.L., Orgogozo V. (2008). The loci of evolution: how predictable is genetic evolution ?. Evolution.

[bib63] Sturmbauer C., Salzburger W., Duftner N., Schelly R., Koblmüller S. (2010). Evolutionary history of the Lake Tanganyika cichlid tribe Lamprologini (Teleostei: perciformes) derived from mitochondrial and nuclear DNA data. Mol. Phylogenet. Evol..

[bib64] Swan B.K., Tupper B., Sczyrba A., Lauro F.M., Martinez-Garcia M., Gonzalez J.M., Luo H., Wright J.J., Landry Z.C., Hanson N.W. (2013). Prevalent genome streamlining and latitudinal divergence of planktonic bacteria in the surface ocean. Proc. Natl. Acad. Sci. U S A.

[bib65] Szendro I.G., Franke J., de Visser J.A.G.M., Krug J. (2013). Predictability of evolution depends nonmonotonically on population size. Proc. Natl. Acad. Sci. U S A.

[bib66] Tenaillon O., Barrick J.E., Ribeck N., Deatherage D.E., Blanchard J.L., Dasgupta A., Wu G.C., Wielgoss S., Cruveiller S., Médigue C. (2016). Tempo and mode of genome evolution in a 50,000-generation experiment. Nature.

[bib67] Toll-Riera M., San Millan A., Wagner A., MacLean R.C. (2016). The genomic basis of evolutionary innovation in Pseudomonas aeruginosa. PLoS Genet..

[bib73] Tripp H.J., Bench S.R., Turk K.A., Foster R.A., Desany B.A., Niazi F., Affourtit J.A., Zehr J.P. (2010). Metabolic streamlining in an open- ocean nitrogen-fixing cyanobacterium. Nature.

[bib68] Wang S., Dai L. (2019). Evolving generalists in switching rugged landscapes. PLoS Comput. Biol..

[bib69] Wang X., Zorraquino V., Kim M., Tsoukalas A., Tagkopoulos I. (2018). Predicting the evolution of Escherichia coli by a data-driven approach. Nat. Commun..

[bib70] Wolf Y.I., Koonin E.V. (2013). Genome reduction as the dominant mode of evolution. Bioessays.

[bib71] Wu G., Yan Q., Jones J.A., Tang Y.J., Fong S.S., Koffas M.A.G. (2016). Metabolic burden: cornerstones in synthetic biology and metabolic engineering applications. Trends Biotechnol..

